# A comprehensive analysis concerning eating behavior associated with chronic diseases among Romanian community nurses

**DOI:** 10.3389/fpubh.2024.1368069

**Published:** 2024-03-21

**Authors:** Lidia-Manuela Onofrei, Maria Puiu, Adela Chirita-Emandi, Costela Lacrimioara Serban

**Affiliations:** ^1^Department of Microscopic Morphology Genetics Discipline, Center of Genomic Medicine, “Victor Babes” University of Medicine and Pharmacy Timisoara, Timisoara, Romania; ^2^Regional Center of Medical Genetics Timis, Clinical Emergency Hospital for Children “Louis Turcanu”, part of ERN ITHACA, Timisoara, Timis, Romania; ^3^Department of Functional Sciences, Discipline of Public Health, “Victor Babes” University of Medicine and Pharmacy Timisoara, Timisoara, Romania; ^4^Department of Functional Sciences, Discipline of Public Health, Center for Translational Research and Systems Medicine, “Victor Babes” University of Medicine and Pharmacy, Timisoara, Romania

**Keywords:** chronic diseases, non-communicable diseases, community nurses, eating behavior, high carbohydrate diet, low carbohydrate diet score, intuitive eating

## Abstract

**Introduction:**

Lifestyle factors, including inadequate eating patterns, emerge as a critical determinant of chronic disease. Apart from caring for patients, nurses should also take an active role in monitoring and managing their own health. Understanding the intricate relationship between nurses’ eating behavior and managing their own health is crucial for fostering a holistic approach to healthcare, therefore our study aimed to evaluate eating behavior and demographic factors influencing chronic disease prevalence in a sample of community nurses from Romania.

**Methods:**

Between October–November 2023, 1920 community nurses were invited to answer an online survey, using an advertisement in their professional network. Of them, 788 responded. In the survey, which included a semi-quantitative food frequency questionnaire with 53 food items, the Intuitive Eating Survey 2 (IES-2), and demographic items were used.

**Results:**

A multivariate model was built for the prediction of the association between eating behavior and other factors associated with chronic diseases. The majority of participants were females (95.1%), with the largest age group falling between 40 and 49.9 years (48.2%). Regarding the EFSA criteria for adequate carbohydrate and fat intake, 20.2% of the group have a high intake of carbohydrates, respectively, 43.4% of the group have a high intake of fat. Analysis of chronic diseases indicated that 24.9% of individuals reported at least one diagnosis by a physician. The presence of chronic disease was associated with a low level of perceived health status, with an OR = 3.388, 95%CI (1.684–6.814), compared to those reporting excellent or very good perceived health status. High stress had an OR = 1.483, 95%CI (1.033–2.129). BMI had an OR = 1.069, 95%CI (1.032–1.108), while low carbohydrate diet score had an OR = 0.956, 95%CI (0.920–0.992). Gender and IES-2 did not significantly contribute to the model, but their effect was controlled.

**Discussion:**

By unraveling the intricate interplay between nutrition, lifestyle, and health outcomes in this healthcare cohort, our findings contribute valuable insights for the development of targeted interventions and support programs tailored to enhance the well-being of community nurses and, by extension, the patients they support.

## 1 Introduction

Lifestyle factors, notably inadequate eating patterns, emerge as pivotal determinants influencing the onset and progression of chronic diseases (1). Inadequate dietary patterns have emerged as significant contributors to the development of obesity and various diseases of civilization, presenting a pressing public health concern globally. These dietary patterns, characterized by excessive intake of energy-dense foods high in sugars, unhealthy fats, and processed ingredients, coupled with insufficient consumption of nutrient-rich whole foods, have been closely linked to the rising prevalence of obesity and overweight populations. Furthermore, such dietary habits are implicated in the onset and progression of chronic diseases such as type 2 diabetes, cardiovascular diseases, hypertension, and certain cancers (2–4).

Nurses play a vital role in chronic disease management, serving as frontline healthcare providers. Their responsibilities encompass patient education, monitoring, and coordination of care to enhance patient outcomes. Nurses often facilitate lifestyle interventions, medication adherence, and provide emotional support to individuals with chronic conditions. Their holistic approach addresses not only the physical aspects of the disease but also the psychological and social components, promoting a patient-centered care model. The collaborative efforts of nurses contribute significantly to the overall well-being and quality of life of individuals living with chronic diseases (5, 6).

Beyond their dedicated roles in patient care, nurses should actively participate in monitoring and managing their own health. Nurses must maintain adequate eating behavior for several reasons. Firstly, nurses play a pivotal role in promoting health, and their own well-being sets an example for others. Secondly, proper nutrition contributes to sustained energy levels, enhancing nurses’ ability to meet the physical and mental demands of their profession. Additionally, adequate eating behavior is linked to overall health, reducing the risk of chronic diseases that could affect job performance. Lastly, nurses with healthy eating habits are better equipped to educate and guide patients on lifestyle choices, creating a positive impact on public health (1, 7, 8).

Recognizing the intricate relationship between nurses’ eating behavior and their overall well-being is paramount for fostering a comprehensive and proactive approach to healthcare (9). In light of this, our study delves into the evaluation of eating behavior and demographic factors influencing chronic disease diagnosis, focusing specifically on a sample of community nurses from Romania. This research aims to unravel the nuanced connections between nurses’ lifestyles, eating habits, and their susceptibility to chronic conditions, offering valuable insights for promoting individual health and advancing healthcare practices.

## 2 Methods

### 2.1 Participants

In Romania, community nurses play a crucial role in providing medical and nursing care to patients outside of traditional hospital settings. Their main objective is to enhance access to quality medico-social services for the population, particularly for vulnerable groups. This involves offering preventive, curative, and recovery medical services and managing complex cases of chronic or rare diseases. Between October–November 2023, out of the 1920 community nurses (90% females) employed in Romania, 788 participants willingly took part in this study, resulting in a response rate of 41.1%.

### 2.2 Procedure

**Figure 1:**
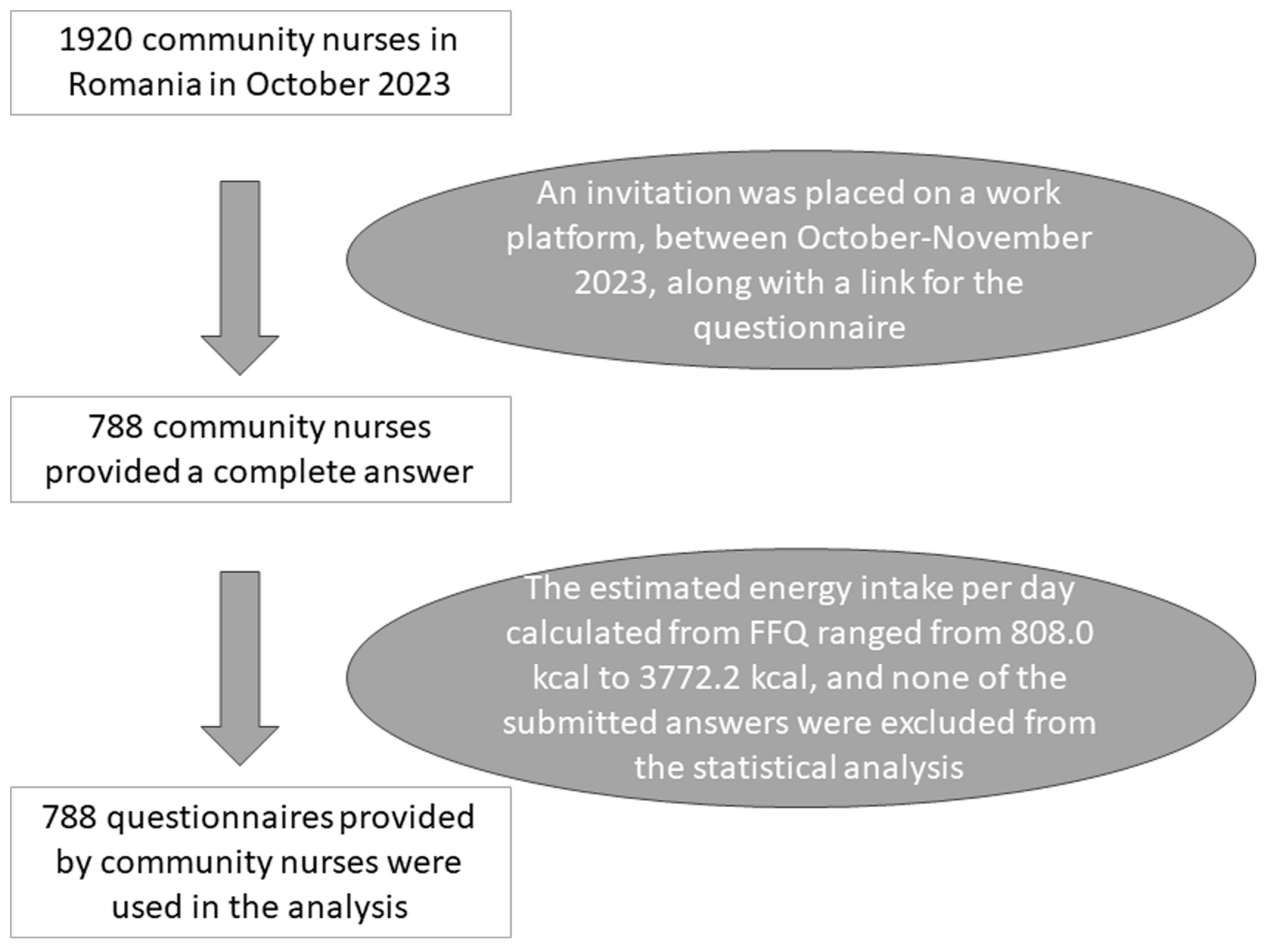
Flow-chart of the recruitment and quality check of questionnaires.

The recruitment of participants for our research project took place in October–November 2023 through an advertisement on a professional network and a national work platform targeting community nurses in Romania. We used the Google Forms platform to embed the questionnaire link in the advertisement. The introductory page of the questionnaire provided details about the study’s purpose, estimated completion time, instruments used, and anticipated outcomes. Participants were required to answer all questions, and their explicit agreement was necessary to gain access to survey. Only those who completed the entire questionnaire were permitted to submit the form. A flow-diagram depicting important aspects of the procedure is presented in Figure 1.

Our study protocol received approval from the Research Ethics Committee of Victor Babes University of Medicine and Pharmacy (Approval No. 30/31.03.2022). Participants voluntarily consented to participate, adhering to the principles outlined in the Helsinki Declaration throughout the study.

#### 2.2.1 Nutritional assessment

Our nutritional assessment involved using a validated FFQ (10, 11), which included 53 different food items. This questionnaire was designed to gather information about food intake over the course of last 30 days. For each item, we investigated the frequency and usual amount of consumption. Additional questions regarding specific items were asked to estimate the quantity of fat or added sugar. The Romanian version of the FFQ is available as a supplementary material (Supplementary material 1). We converted the intakes to grams using household scale guidelines (12) and calculated energy and macronutrient intakes for each individual using a specialized computer program. Finally, we transformed macronutrient intakes into a percentage of contribution to the total energy consumed.

To determine adherence to a low carbohydrate diet, a score called the low carbohydrate diet (LCD) score was calculated, using deciles of the percentage of macronutrients (13, 14). The score for carbohydrates ranged from one to ten, with the lowest decile receiving the highest score of 10, and the highest decile receiving the lowest score of one. For fat and protein, the lowest decile received a score of one, and the highest decile received a score of 10. The individual scores for each macronutrient were then added to get the LCD score, which can range from 3 to 30, with higher scores indicating better adherence to a low carbohydrate diet. European Food and Safety Authority (EFSA) recommendations (15) for adequate intake, were used to create two variables for individual meeting or not the recommended intake for carbohydrates and fat, using the contributing percentage of each of the macronutrients to total energy intake.

The Intuitive Eating Scale-2 (IES-2) is the most widely used measure to assess intuitive eating (16) and was recently validated in Romanian (17). It consists of 23 items. Items 4, 5, 6, 11–23 are scored Strongly Disagree = 1, Disagree = 2, Neutral = 3, Agree = 4, Strongly Agree = 5 and for items 1, 2, 3, 7, 8, 9, and 10 the scoring is reversed. The IES-2 score is obtained by adding all the individual item scores and then by division to the number of items (23 items). Higher scores on the IES-2 indicate a greater ability to eat intuitively. The Romanian version of the IES-2 is available as a supplementary material (Supplementary material 2).

In addition to the aforementioned instruments, the study also included questions regarding gender, age, relationship status (5 categories), education level, using International Standard Classification of Education (ISCED) (18), self-perceived health status (5 levels), years of experience as a community nurse, and self-perceived stress levels. Stress levels were rated on a 10-point scale, with higher values indicating higher levels of stress. Moreover, community nurses were requested to assess their height and weight through measurement, as well as to indicate the existence of chronic diseases and provide diagnoses of such diseases, which were set by physicians. Participants were presented with a dropdown list of seven chronic diseases that have a significant impact on public health and are heavily influenced by lifestyle choices (1). These diseases included hypertension, heart failure, ischemic heart disease, type 2 diabetes, obesity, asthma, and chronic obstructive pulmonary disease. Additionally, participants were given the option to provide an open response and list any other chronic diseases that have been diagnosed by their physicians. For those who reported one or more diagnoses, a follow-up question was asked regarding their treatment of the chronic disease (s).

### 2.3 Data management and statistics

Data transformations and statistical analyses were performed using IBM SPSS version 21. All submitted participant responses were included in the analysis. Height and weight were self-reported, but nurses were asked to report a measurement not older than 3 months. Height and weight were further used for the calculation of body mass index (BMI; BMI = weight (kg)/height (m)^2^). The nutritional status of the participants was determined using the following BMI thresholds: underweight (BMI below 18.5 kg/m^2^), normal weight (18.5–24.9 kg/m^2^), overweight (25–29.9 kg/m2) and obese (over 30 kg/m^2^). Other variables were categorized as such: age was split into 3 groups: <=40 years, 40–49.9 years and > =50 years; relationship status was dichotomized: in a relationship versus alone; education was divided into 2 levels, equivalent to ISCED 4 or less, ISCED 5 or above; self-perceived health status was categorized into 2 levels: excellent or very good health status vs. lower levels of perception; years of experience as a community nurse were split into 3 groups: <5 years, 5–9.9 years and > =10 years; self-perceived stress levels were split into tertiles and highest tertile was used to define the variable high levels of stress.

Normal distribution was tested using the Kolmogorov-Smirnoff test. Mean and standard deviation (SD) were used to present continuous variables. Absolute and relative frequencies were used for categorical variables. Statistical significance was determined by a *p*-value < 0.05. To compare means, ANOVA with Sidak correction was utilized. To compare proportions, the Mann–Whitney test was used for the comparison of 2 factors, and the Kruskal–Wallis test was used for the comparison of 3 factors. Bonferroni correction was used to assess the statistical significance when multiple Mann–Whitney tests were applied. Demographic and nutritional intake quality data, including LCD score and intuitive eating score, were used in a regression analysis model to determine the association with the presence of at least a chronic disease diagnosis established by a physician.

## 3 Results

Out of the sample, the majority were females, making 95.1% (749). The largest age group in the sample was 40–49.9 years old, consisting of 380 individuals (48.2%). A significant proportion of the nurses, 36.5% had worked as community nurses for more than 10 years, while 32.7% had worked between 5–9.9 years. The mean age of the whole group was 43.4 years with a standard deviation of 7.8 years, ranging from 22 to 65 years old. On average, the group had worked as community nurses for 8 years, ranging from 0 to 22 years (Table 1).

**Table 1:** Descriptive statistics of the sample of community nurses per presence of a diagnosis of chronic disease and total (*N* = 788).

Variables		Diagnosis of chronic disease		*p*-value	Total
		No	Yes		
Gender*	Male	29 (4.9%)	10 (5.2%)	0.834	39 (4.9%)
	Female	568 (95.1%)	181 (94.8%)		749 (95.1%)
Age categories*	<=40 years	230 (38.5%)	32 (16.8%)	<0.001	262 (33.2%)
	40–49.9 years	282 (47.2%)	98 (51.3%)		380 (48.2%)
	> = 50 years	85 (14.2%)	61 (31.9%)		146 (18.5%)
Work as a community nurse*	<5	201 (33.7%)	41 (21.5%)	<0.001	242 (30.7%)
	5–9.9 years	201 (33.7%)	57 (29.8%)		258 (32.7%)
	> = 10 years	195 (32.7%)	93 (48.7%)		288 (36.5%)
Excellent or very good perceived health status*	No	483 (80.9%)	181 (94.8%)	<0.001	664 (84.3%)
	Yes	114 (19.1%)	10 (5.2%)		124 (15.7%)
With partner*	No	74 (12.4%)	21 (11.0%)	0.605	95 (12.1%)
	Yes	523 (87.6%)	170 (89.0%)		693 (87.9%)
Education ISCED 4 or less*	No	157 (26.3%)	41 (21.5%)	0.180	198 (25.1%)
	Yes	440 (73.7%)	150 (78.5%)		590 (74.9%)
BMI categories*	Underweight	14 (2.3%)	3 (1.6%)	<0.001	17 (2.2%)
	Normal weight	273 (45.7%)	50 (26.2%)		323 (41.0%)
	Overweight	208 (34.8%)	78 (40.8%)		286 (36.3%)
	Obese	102 (17.1%)	60 (31.4%)		162 (20.6%)
Age (years)**	Mean (SD)	42.3 (7.7)	47.1 (7.0)	<0.001	43.4 (7.8)
Worked as a community nurse (years)**	Mean (SD)	9.1 (6.6)	11.3 (6.5)	<0.001	8.0 (6.6)
Stress level (1 to10)*	Mean (SD)	5.2 (2.6)	5.9 (2.5)	0.001	5.4 (2.6)
BMI (kg/m^2^)**	Mean (SD)	25.9 (4.7)	28.4 (5.4)	<0.001	26.5 (5.0)

*Mann–Whitney test, **t-test.

Regarding personal status and education, most of the nurses were in a relationship, comprising 87.9% or 693 individuals, and 74.9% had obtained at most an ISCED 4 diploma. Only 15.7% or 124 individuals reported an excellent or very good perceived health status. The mean BMI of the group was 26.5 kg/m^2^. Among the participants, 36.3% were overweight, 20.6% were classified as obese, 2.2% were underweight and 41.0% had a normal weight (Table 1). Using the presence/absence of chronic disease as a factor, no significant differences were found between the different categories in the following variables: gender (*p* = 0.834), social status (*p* = 0.605), and education level (*p* = 0.180). However, the group with the chronic disease did exhibit higher age [47.1 (7.0) vs. 42.3 (7.7)] and experience as community nurses [11.3 (6.5) vs. 9.1 (6.6)], than those without chronic disease. Additionally, the group with chronic diseases reported greater stress levels and perceived their health status to be lower. In terms of nutrition status, those with chronic disease had a higher reported BMI of 28.4 (5.4) kg/m^2^, than those without chronic disease which had a mean of 25.9 (4.7) kg/m^2^, the difference being significant (Table 1).

In terms of chronic diseases, 24.9% or 191 individuals reported having at least one diagnosed by a physician. Among all community nurses, 16.5% had one chronic disease, 4.6% had two, and 3.2% had three or more. The community nurses reported several diagnoses of chronic diseases, with hypertension being the most prevalent at 9.8% (77 cases). This was followed by obesity at 4.4% (35 cases), thyroid diseases at 3.8% (30 cases), and dyslipidemia at 3.7% (29 cases). Type II diabetes had a prevalence of 2.2% with 17 cases, while heart failure and ischemic heart disease recorded 1.9% (15 cases) and 1.6% (13 cases) point prevalence, respectively. Asthma had a point prevalence of 1.4% (11 cases), while other diseases had significantly lower point prevalence (data not shown).

In regards to chronic disease treatment, it is noteworthy that only 22 out of 191 individuals (11.5%) who reported a chronic condition did not adhere to their prescribed treatment plan. Interestingly, all of these non-adherers reported only a single chronic condition. Additionally, it is worth mentioning that for both heart failure and ischemic heart disease, all patients followed their prescribed treatment plan.

### 3.1 Eating behavior evaluation

The mean (SD) LCD score was 16.5 (6.8) with a range from a minimum of 3 and a maximum of 29 points. The mean (SD) IES-2 score, on the other hand, was 3.3 (0.3), ranging from a minimum of 1.91 to a maximum of 4.13 points. Energy intake ranged from a minimum of 808.0 kcal to a maximum of 3772.2 kcal, with a mean and a standard deviation of 1917.9 (612.6) kcal. In terms of EFSA’s recommended intake, 481 individuals, or 61%, meet the suggested proportion of carbohydrate intake, while 56.6% of the overall group meet the recommended proportion of fat intake. However, it’s worth noting that 18.8% of the group, or 148 individuals, have a low intake of carbohydrates, while 20.2% of the group, or 159 individuals, have a high intake of carbohydrates. Additionally, 43.4% of the group have a high intake of fat.

Based on the LCD score tertiles, the medium carbohydrate intake group shows a significantly higher IES-2 score (*p* < 0.001), but only when compared to the high carbohydrate group. Additionally, the medium carbohydrate group features the highest proportions of EFSA recommended carbohydrate intake, while the high carbohydrate group has the highest proportion of recommended fat intake (*p* < 0.001). Those in the high carbohydrate intake group have a higher prevalence of chronic diseases (*p* = 0.001), while those in the low carbohydrate intake group have a higher prevalence of excellent or very good perceived health status (*p* = 0.002). It’s worth noting that education level (*p* = 0.649), high-stress level (*p* = 0.127), total energy intake (*p* = 0.960), age (*p* = 0.227), experience as a community nurse (*p* = 0.568), and BMI (*p* = 0.983) are not influenced by the tertiles of LCD score (Table 2).

**Table 2:** Macronutrient intake and demographic variables by tertiles of LCD score (*N* = 788).

Eating behavior and demographic variables		Tertiles of LCD score			
		High carbohydrate intake	Medium carbohydrate intake	Low carbohydrate intake	*p*-value
Carbohydrate recommended intake n (%)*	Low	0 (0.0%)	10 (4.5%)	138 (47.8%)	<0.001
	recommended	117 (42.5%)	214 (95.5%)	150 (51.9%)	
	high	158 (57.5%)	0 (0.0%)	1 (0.3%)	
Fat recommended intake n (%)*	low	0 (0.0%)	0 (0.0%)	0 (0.0%)	<0.001
	recommended	260 (94.5%)	142 (63.4%)	44 (15.2%)	
	high	15 (5.5%)	82 (36.6%)	245 (84.8%)	
Chronic diseases diagnosed by physician n (%)*	No	189 (31.7%)	171 (28.6%)	237 (39.7%)	0.001
	Yes	86 (45.0%) ^a^	53 (27.7%) ^b^	52 (27.2%) ^b^	
Excellent or very good perceived health status n (%)*	No	241 (36.3%)	187 (28.2%)	236 (35.5%)	0.002
	Yes	34 (27.4%) ^a^	37 (29.8%) ^a^	53 (42.7%) ^b^	
High perceived stress level n (%)*	No	156 (32.5%)	147 (30.6%)	177 (36.9%)	0.127
	Yes	119 (38.6%)	77 (25.0%)	112 (36.4%)	
Education ISCED 4 or less n (%)*	No	64 (23.3%)	60 (26.8%)	74 (25.6%)	0.649
	Yes	211 (76.7%)	164 (73.2%)	215 (74.4%)	
IES-2 score mean (SD)**		3.24 (0.33) ^a^	3.32^b^ (0.27)	3.31 (0.28) ^b^	0.002
Energy intake (Kcal) mean (SD)**		1925.7 (653.5)	1910.1 (603.8)	1916.6 (580.2)	0.960
Age (years) mean (SD) **		43.87 (7.80)	43.82 (8.06)	42.74 (7.68)	0.227
Experience as a Community nurse (years) mean (SD) mean (SD) **		10.04 (7.51)	9.02 (5.86)	9.61 (6.30)	0.568
BMI (kg/m^2^) mean (SD) **		26.61 (5.25)	26.50 (4.73)	26.51 (4.91)	0.983

LCD, low carbohydrate diet score; IES-2, Intuitive Eating Scale 2; ISCED, International Standard Classification of Education; SD, standard deviation.

*Kruskal-Wallis test followed if significant by Mann Whitney tests with Bonferroni correction for multiple comparisons **ANOVA with Sidak adjustment ^a,b^ similar superscript letters denote no statistically significant between groups. Different superscript letters denote significant differences between groups, after Bonferroni or respective Sidak correction.

When dividing participants into tertiles based on their IES-2 scores, we found that the recommended carbohydrate intake proportions were similar across all groups (*p* = 0.331). However, those who achieved the recommended fat intake were more prevalent in the top tertile of intuitive eating (*p* = 0.045). Furthermore, individuals in the highest tertile tended to perceive their health status as excellent or very good (*p* < 0.001), had greater experience as a community nurse (*p* = 0.003), were older (*p* = 0.002), had a lower BMI (*p* < 0.001) and had lower energy intake (*p* < 0.001) than those in the lower tertiles. The presence of chronic disease, level of education, and high-stress levels remained consistent across all IES-2 tertiles (Table 3).

**Table 3:** Macronutrient intake and demographic variables by tertiles of IES-2 score (*N* = 788).

Eating behavior and demographic variables		Tertiles of IES-2 score			
		Low tertile	Medium tertile	High tertile	*p*-value
Carbohydrate recommended intake n (%)*	low	51 (19.0%)	51 (18.6%)	46 (18.7%)	0.331
	recommended	151 (56.3%)	170 (62.0%)	160 (65.0%)	
	high	66 (24.6%)	53 (19.3%)	40 (16.3%)	
Fat recommended intake n (%)*	low	0 (0.0%)	0 (0.0%)	0 (0.0%)	0.045
	recommended	140 (52.2%)	152 (55.5%)	154 (62.6%)	
	high	128 (47.8%)	122 (44.5%)	92 (37.4%)	
Chronic diseases diagnosed by physician n (%)*	No	208 (77.6%)	196 (71.5%)	193 (78.5%)	0.127
	Yes	60 (22.4%)	78 (28.5%)	53 (21.5%)	
Excellent or very good perceived health status n (%)*	No	234 (87.3%)	245 (89.4%)	185 (75.2%)	<0.001
	Yes	34 (12.7%) ^a^	29 (10.6%) ^a^	61 (24.8%) ^b^	
High perceived stress level n (%)*	No	157 (58.6%)	168 (61.3%)	155 (63.0%)	0.582
	Yes	111 (41.4%)	106 (38.7%)	91 (37.0%)	
Education ISCED 4 or less n (%)*	No	54 (20.1%)	73 (26.6%)	71 (28.9%)	0.058
	Yes	214 (79.9%)	201 (73.4%)	175 (71.1%)	
Energy intake (Kcal) mean (SD)**		2008.1 (623.7) ^a^	1959.8 (620.3) ^a^	1773.0 (566.4) ^b^	<0.001
Age (years) mean (SD)**		42.04 (7.84) ^a^	44.23 (7.45) ^b^	44.08 (8.08) ^b^	0.002
Experience as a Community nurse (years) mean (SD)		8.46 (5.81) ^a^	10.31 (6.53) ^b^	10.02 (7.42) ^b^	0.003
BMI (kg/m^2^) mean (SD)**		27.40 (5.26) ^a^	26.86 (4.99) ^a,b^	25.26 (4.37) ^b^	<0.001

IES-2 Intuitive Eating Scale 2, ISCED—International Standard Classification of Education, SD—standard deviation, *Kruskal-Wallis test followed if significant by Mann Whitney tests with Bonferroni correction for multiple comparisons **ANOVA with Sidak adjustment ^a,b^similar superscript letters denote no statistically significant between groups. Different superscript letters denote significant differences between groups, after Bonferroni or respective Sidak correction.

### 3.2 Prediction model for chronic disease

We used a prediction model to analyze the connection between demographic variables, IES-2, and LCD score concerning the diagnosis of at least one chronic disease, set by a physician. Table 4 displays the OR and 95% confidence intervals of each predictor fitted in the model. Our findings indicate that a low level of perceived health status has the strongest association with the presence of diagnosis of chronic diseases, with an OR of 3.388, 95%CI 1.684–6.814, compared to those reporting excellent or very good perceived health status. High stress has an OR of 1.483, 95%CI 1.033–2.129. BMI had an OR of 1.069, 95%CI 1.032–1.108, while LCD score had an OR of 0.956, 95%CI 0.920–0.992. While other social demographic factors did not significantly contribute to the model, they were still taken into account (Table 4).

**Table 4:** Prediction model for the association of demographic and eating habits to the presence of a diagnosis of chronic disease (*N* = 788).

Factors	OR	95% confidence interval for OR	
		Lower	Upper
Gender (Male vs. Female)	0.930	0.408	2.119
High Stress (Yes vs. No)	1.483	1.033	2.129
IES-2 score	0.858	0.464	1.586
LCD score	0.969	0.944	0.995
Excellent or very good perceived health status (No vs. Yes)	3.388	1.684	6.814
BMI (kg/m^2^)	1.069	1.032	1.108
Age (years)	1.089	1.062	1.117
Constant	0.001		

Logistic regression chi-square = 112.3, degrees of freedom = 7, *p* < 0.001, Independent variable: Diagnosis of at least one chronic disease. Independent variables: Gender (Male vs Female), High Stress (Yes vs No), IES-2 score, LCD score, Excellent or very good perceived health status (Yes vs No), BMI (kg/m2), Age (years).

## 4 Discussion

To the best of our knowledge, this is the first study to shed light on the eating habits concerning chronic diseases in community nurses from Romania. This assessment is important for future programs targeting improvement in eating habits in community nurses, with personal benefits, but also for their patients’ benefits, because being able to provide nutrition screening and appropriate nutrition advice is essential to improve healthy eating and subsequent health outcomes of their patients.

The trend of chronic diseases in Europe is marked by a significant rise in prevalence, posing substantial challenges to healthcare systems. Lifestyle factors, including sedentary behavior and poor dietary habits, contribute to the increasing burden of diseases such as diabetes, cardiovascular conditions, and obesity. Aging populations further amplify this trend, as chronic diseases are often more prevalent in older demographics (19, 20). In our analysis, we found that among individuals with an age range from 22 to 65 years, the point prevalence of any chronic disease was 24.2%, and for those with more than two chronic diseases, it was 7.8%.

This increase in multimorbidity among adults under the age of 65 challenges the traditional perception that chronic conditions primarily affect older populations. Overall, the prevalence of multimorbidity across Europe is estimated to be 39.2% with a 95% confidence interval of 33.2–45.2%, with higher prevalences in Europe in women 43.4% (24.8–50.0), compared to men 37.4% (31.7–55.1) (21). The burden of multimorbidity not only impacts individual well-being but also poses significant challenges to healthcare systems in terms of management and resource allocation (21, 22).

According to our study, the predictive model for the association between demographics and eating habits with chronic disease diagnosis found that individuals diagnosed with chronic disease tend to experience higher levels of stress, perceive their health status as poor, have an older age and higher BMI, and consume more carbohydrates compared to those without a diagnosis.

Individuals diagnosed with chronic conditions often report a lower perceived health status, reflecting the multifaceted impact of these conditions on well-being. The psychological and emotional burden associated with chronic diseases can contribute to a negative perception of overall health (23). Conversely, those with a positive perception of health may exhibit better coping mechanisms and adherence to healthcare recommendations. Understanding the interplay between chronic diseases and subjective health assessments is crucial for comprehensive health management and targeted interventions (24).

While age is not a modifiable factor, factors such as high levels of stress, high BMI, and low food intake quality can be improved. Incorporating mindfulness practices, like meditation and deep breathing exercises, can effectively reduce stress levels, promoting mental well-being (25, 26). The management of BMI can include practicing a regular exercise routine, including both aerobic and strength-training exercises, which are a key strategy for promoting weight loss and improving overall physical health (27). Interventions providing education plus personalized advice and feedback empower individuals to make healthier food choices, emphasizing a balanced diet rich in fruits, vegetables, whole grains, and lean proteins to enhance overall food quality (28). Behavioral interventions, such as goal-setting and self-monitoring, can foster sustainable lifestyle changes, aiding in stress reduction, BMI management, and the adoption of healthier eating habits (1). Systemic interventions to improve walkability, access to healthy food and sport facilities are equally important (29).

Our investigation of the eating habits of community nurses analyzed both the quality of their food intake using the Low Carbohydrate Diet (LCD) score, which is a score based on deciles of macronutrients, as well as their intuitive eating habits with the Intuitive Eating Score 2 (IES-2) index. Our findings revealed that community nurses with a high carbohydrate intake had a much higher prevalence of chronic disease (45.0%) compared to those in the middle (27.7%) and low carbohydrate diet tertiles (27.2%), but no effect was found between LCD score and BMI.

Low-carbohydrate diets have gained popularity due to their effectiveness in achieving short-term weight loss, which is further enhanced when coupled with high-protein diets (30, 31). However, recent studies have yielded inconsistent findings regarding their long-term impact on diabetes (32, 33). Though useful and popular for short-term weight loss and short-term reduction in cardiovascular risk factors (34), diets low in carbohydrates and high in fat, especially saturated fat put a supplementary risk for all-cause mortality (33–35). On the other hand, increased consumption of dietary carbohydrate intake is associated with an increased risk of cardiovascular disease, the risk increasing by 1.02 times for every 5% rise in dietary carbohydrate consumption (36). The ARIC cohort showed a U-shaped all-cause mortality, with the lowest risk of mortality in diets with 50–55% energy from carbohydrates (37). The source of fat and protein further modulated the increased mortality in high and low percentages of energy from carbohydrates, with plant sources having a protective effect and animal-derived fat or protein having a detrimental effect on all-cause mortality (37).

Intuitive eating is a holistic approach to nutrition that prioritizes one’s body cues over external diet rules. By listening to and trusting internal signals, such as hunger and fullness, one can develop a mindful and non-restrictive relationship with food that promotes overall well-being and a healthier relationship with eating. Recent meta-analyses have found that those who practice intuitive eating tend to have a lower BMI (38) and a higher quality diet (39). In our study, higher levels of intuitive eating were linked to lower BMI levels and lower energy intake and higher percentage of adequate intake of fat. We discovered that nurses in the highest tertile of intuitive eating had significantly lower BMI values of 25.26 (4.37), compared to those in the lowest tertile of intuitive eating, who had BMI values of 27.40 (5.26). Additionally, energy intake was found to be associated with tertiles of intuitive eating score, with the highest tertile of intuitive eating being linked to lower energy intake with a mean of 1773.0 (566.4) kcal when compared to other tertiles 2008.1 (623.7) kcal, respective 1959.8 (620.3) kcal.

Our research has shown that community nurses with more age and experience tend to have higher intuitive scores. This may be attributed to the fact that as individuals age, they develop a greater understanding of their body and personal preferences, leading to a more refined and knowledgeable approach to intuitive eating. The principles of intuitive eating are in line with mindfulness techniques, which encourage individuals to be fully present and aware of their eating habits (40). Our investigation did not uncover any previous studies utilizing the Intuitive Eating Scale 2 (IES-2) specifically among nurses.

Nurses often experience high levels of occupational stress due to demanding workloads, long hours, and the emotionally charged nature of their profession, impacting their mental well-being and work performance (41, 42). Chronic stress triggers physiological responses that can contribute to the development and exacerbation of chronic diseases, including cardiovascular conditions and metabolic disorders (43, 44). Stress often influences unhealthy coping behaviors, such as poor dietary choices and lack of exercise, which are linked to the development of chronic conditions like obesity and diabetes (43, 45). The interplay between chronic stress and chronic diseases forms a complex cycle, where stress exacerbates the conditions, and the presence of chronic diseases can, in turn, amplify stress levels, highlighting the importance of holistic approaches to health management (44).

The Body Mass Index (BMI) analysis in Europe reveals an increasing prevalence of overweight and obesity across diverse populations (46, 47). Elevated BMI is closely linked to the rising incidence of chronic diseases, including diabetes, cardiovascular conditions, and certain cancers. The NCD Risk Factor Collaboration reports a mean BMI of 27.3 (26.3–27.7) kg/m^2^ for Romania, which is comparable to our sample’s BMI of 26.5 (5.0) kg/m^2^. The prevalence of obesity in the community nurse cohort, measured by weight and height, is 20.6% and the prevalence of combined overweight and obesity is 56.9%, consistent with other estimates (48). There is a significant difference in the prevalence of the diagnosis of obesity reported by nurses in the chronic diseases section of just 4.4% and the prevalence obtained from measurements of 20.6%. The missed diagnosis of obesity remains a prevalent concern in healthcare, as it often goes unrecognized due to societal misconceptions and biases. Healthcare providers may not consistently assess or address obesity during routine examinations, leading to underdiagnosis in both adult and child populations (49–51). The consequences of missed obesity diagnoses include delayed intervention and increased risk for associated health conditions such as diabetes, cardiovascular diseases, and mental health issues (52, 53).

According to our study, hypertension exhibited the highest point prevalence (9.8%) among chronic reported diseases. The prevalence of hypertension (in males and females) has doubled worldwide between 1990 and 2019 (54). According to the same review (54), in Romania, the prevalence of hypertension is 43.9% (with 95% CI 35.1–52.8) with a detection awareness of 74.6% (with 95%CI 61.8–85.4). The alarming increase in hypertension diagnosis highlights an unsettling trend in multimorbidity. Hypertension frequently acts, along with heart diseases, and diabetes, as a primary contributor in later multimorbidity (55).

Treatment non-adherence in our group was 11.5% of all patients with a chronic disease. Non-adherence to treatment poses a significant risk to multimorbidity, as individuals with chronic conditions may experience worsening health outcomes when failing to follow prescribed medical plans. The complex interplay of multiple chronic diseases can exacerbate if treatments are not consistently adhered to, leading to increased morbidity and complications (56, 57). Poor adherence to medication regimens, lifestyle modifications, and regular health check-ups may contribute to the progression and development of additional chronic conditions. Addressing non-adherence is crucial in preventing the cascade of health issues associated with multimorbidity, highlighting the importance of patient education, support systems, and healthcare interventions to promote treatment compliance (1, 55).

Our study has certain limitations, such as self-reporting bias and selection bias. The self-reported bias pertains not only to the nutrition evaluation, but also to the reported diagnosis of chronic disease. It is important to note that the design of this study was cross sectional, which means that no causality can be established between eating patterns and the presence of chronic diseases. Instead, the findings can only be interpreted as associations. Although the response rate of our study was good, it is common knowledge that individuals who participate in nutrition studies are typically more interested in nutrition and healthy living than those who do not participate. There is a predominantly female cohort, however this is concordant with the prevalence of female community nurses in Romania.

The present study on the eating habits and health profiles of community nurses in Romania provides comprehensive insights, which may serve as a foundation for future research endeavors. Given the findings of this study, several key areas warrant further attention. Longitudinal studies could clarify on the dynamic nature of dietary patterns, stress levels, and chronic disease diagnoses among nurses over time, thereby facilitating a deeper understanding of their interchange. Moreover, comparative research across diverse healthcare settings and regions could provide valuable insights into the factors influencing these outcomes.

Additionally, intervention studies targeting modifiable factors identified in this research, such as stress reduction techniques, healthy weight management strategies, and nutritional education programs, could be explored to assess their effectiveness in promoting overall well-being among healthcare professionals. Such research would not only benefit the healthcare professionals but also the patients they serve. It is widely acknowledged that the health of healthcare professionals is directly linked to the quality of care that patients receive, and therefore, interventions that improve the health and well-being of healthcare professionals can have a positive impact on patient outcomes (58, 59).

Lastly, collaborative efforts between healthcare institutions, policymakers, and researchers are essential to develop and implement tailored programs that prioritize the health and resilience of nurses, thereby contributing to the mitigation of the burden of chronic diseases within healthcare systems. The implementation of such programs would require a concerted effort and the development of strategies that are feasible, sustainable, and evidence-based. Overall, these research endeavors can contribute significantly to the advancement of knowledge in this field and ultimately lead to positive health outcomes for healthcare professionals and patients alike.

## 5 Conclusion

This study provides a comprehensive insight into the eating habits and health profiles of community nurses in Romania, uncovering noteworthy associations between dietary patterns, stress levels, and chronic disease diagnoses. The prevalence of chronic diseases among this population, particularly hypertension, underscores the importance of targeted interventions for healthcare professionals. Notably, the study identifies modifiable factors, including stress, BMI, and dietary habits based on high carbohydrate diets, offering potential avenues for personalized health interventions. The findings emphasize the need for holistic strategies, encompassing stress reduction, healthy weight management, and nutritional education to enhance overall well-being. As healthcare systems struggle with the increasing burden of chronic diseases, these insights contribute to the development of tailored programs that prioritize the health and resilience of healthcare providers.

## Data availability statement

The raw data supporting the conclusions of this article will be made available by the authors, without undue reservation.

## Ethics statement

The studies involving humans were approved by the Research Ethics Committee of Victor Babes University of Medicine and Pharmacy. The studies were conducted in accordance with the local legislation and institutional requirements. The participants provided their written informed consent to participate in this study.

## Author contributions

L-MO: Conceptualization, Investigation, Writing – original draft, Methodology. MP: Conceptualization, Data curation, Investigation, Methodology, Writing – review & editing. AC-E: Conceptualization, Data curation, Methodology, Writing – review & editing. CS: Conceptualization, Data curation, Formal analysis, Methodology, Writing – original draft.
